# Sensory- and Action-Oriented Embodiment of Neurally-Interfaced Robotic Hand Prostheses

**DOI:** 10.3389/fnins.2020.00389

**Published:** 2020-05-07

**Authors:** Giovanni Di Pino, Daniele Romano, Chiara Spaccasassi, Alessandro Mioli, Marco D’Alonzo, Rinaldo Sacchetti, Eugenio Guglielmelli, Loredana Zollo, Vincenzo Di Lazzaro, Vincenzo Denaro, Angelo Maravita

**Affiliations:** ^1^Research Unit of Neurophysiology and Neuroengineering of Human-Technology Interaction (NeXTlab), Università Campus Bio-Medico di Roma, Rome, Italy; ^2^Psychology Department & NeuroMi, Milan Center for Neuroscience, University of Milan-Bicocca, Milan, Italy; ^3^National Institute for Insurance Against Accidents at Work, Bologna, Italy; ^4^Research Unit of Advanced Robotics and Human-Centred Technologies, Università Campus Bio-Medico di Roma, Rome, Italy; ^5^Research Unit of Neurology, Neurophysiology, Neurobiology, Università Campus Bio-Medico di Roma, Rome, Italy; ^6^Research Unit of Orthopedics and Traumatology, Università Campus Bio-Medico di Roma, Rome, Italy

**Keywords:** neural interface, sensory feedback, robotic hand prostheses, embodiment, multisensory integration

## Abstract

Embodiment is the percept that something not originally belonging to the self becomes part of the body. Feeling embodiment for a prosthesis may counteract amputees’ altered image of the body and increase prosthesis acceptability. Prosthesis embodiment has been studied longitudinally in an amputee receiving feedback through intraneural and perineural multichannel electrodes implanted in her stump. Three factors—invasive (vs non-invasive) stimulation, training, and anthropomorphism—have been tested through two multisensory integration tasks: visuo-tactile integration (VTI) and crossing-hand effect in temporal order judgment (TOJ), the former more sensible to an extension of a safe margin around the body and the latter to action-oriented remapping. Results from the amputee participant were compared with the ones from healthy controls. Testing the participant with intraneural stimulation produced an extension of peripersonal space, a sign of prosthesis embodiment. One-month training extended the peripersonal space selectively on the side wearing the prostheses. More and less-anthropomorphic prostheses benefited of intraneural feedback and extended the peripersonal space. However, the worsening of TOJ performance following arm crossing was present only wearing the more trained, despite less anthropomorphic, prosthesis, suggesting that training was critical for our participant to achieve operative tool-like embodiment.

## Introduction

Despite improved mechatronic features have made hand prostheses more dexterous, their abandonment exceeds 30% (Cordella et al., 2016). Besides high weight and cost, low life-like appearance, low comfort and dexterity (Biddiss and Chau, 2007), and difficult pre-prosthetic training (Peerdeman et al., 2011), what amputees claim as a main limitation is that, regardless of the level of prosthesis functionality, they perceive it as an “inert supplement” or an “extracorporeal structure” and not as part of their body (Scarry, 1994).

As a multipurpose tool allowing capabilities and conveying sensory inflows, the hand is located at the human-environment frontier and defines the boundary of the body, so that its loss greatly affects how amputees perceive themselves and their body (Drench, 1994; Flannery and Faria, 1999). Somatosensory feedback plays a key role in dexterous manipulation and boosts motor learning. Today, commercial hand prostheses do not offer sensory feedback, although position and force information from the prosthesis are identified as design priority for myoelectric devices, in order to increase acceptance (Biddiss and Chau, 2007).

Those reasons generated a strong research effort to develop and test prosthesis-user interfacing systems that, in parallel to a better motor control, can offer rich and pleasant feedback. Sensory feedback from hand prostheses have been delivered employing intraneural (Dhillon and Horch, 2005; Rossini et al., 2010; Page et al., 2018; Petrini et al., 2018) and perineural (Ortiz-Catalan et al., 2014; Tan et al., 2014) electrodes, targeted sensory reinnervation (Kuiken et al., 2007; Marasco et al., 2009; Vadalà et al., 2017), or non-invasive sensory substitution (Antfolk et al., 2013). Richer sensory feedback from the prosthesis showed to improve motor ability (Valle et al., 2018; Zollo et al., 2019), object features discrimination (Raspopovic et al., 2014), and to counteract amputation-induced maladaptive brain plasticity (Di Pino et al., 2014; Serino et al., 2017).

Convergent multisensory afference build the representation of the body in the brain, which has been shown to be flexible to the point of integrating external objects not belonging to the self; the perceptual process producing such integration is known as embodiment (Iriki et al., 1996; Maravita and Iriki, 2004). Thus, a prosthesis enabling a more physiological sensory feedback is expected to greatly improve its embodiment and acceptance (Murray, 2004; Svensson et al., 2017; Graczyk et al., 2019).

Few reports tested the embodiment of worn prostheses able to deliver sensory feedback in amputees with targeted sensory reinnervation (Marasco et al., 2018), perineural flat interface nerve electrodes (FINE) (Schiefer et al., 2016; Graczyk et al., 2018), and with intraneural electrodes (Page et al., 2018; Rognini et al., 2018; Valle et al., 2018). All of them employed the rubber hand illusion (RHI) paradigm (Botvinick and Cohen, 1998) or modified versions of it (Page et al., 2018; Rognini et al., 2018), or interviewed participants using questionnaires derived from the one typically employed in the RHI.

However, the translation of RHI findings to prosthesis embodiment in amputees is not free from possible pitfalls; RHI setup is very structured and artificial, the fake hand is not worn and typically cannot move, and the illusion is only temporary (D’Alonzo et al., 2020; Niedernhuber et al., 2018).

Easily gathered clues on embodiment also come from changes reported by the subject in the perceived length of the phantom limb (Rossini et al., 2010; Graczyk et al., 2018; Rognini et al., 2018; Valle et al., 2018). Still, body representation and phantom awareness are very likely different concepts, partly relying on different brain networks (Ehrsson et al., 2005; Flor et al., 2006; Reilly and Sirigu, 2008; Di Pino et al., 2009; Kikkert et al., 2019).

Any outer event is caught by different sensory modalities; their flow of information is integrated by our brain to have a high probability of experiencing the fact with accuracy. The way the brain integrates senses while experiencing an event depends on the relation of that event with our body. For instance, our brain integrates faster somatosensory and visual stimuli delivered close to the body, within the so-called peripersonal space (PPS) (Fogassi et al., 1996). PPS is the space around the body that can be directly acted upon by the body and where the analysis of external events is more critical to ensure efficient action and protection against threat (Clery et al., 2015b; de Vignemont and Iannetti, 2015). Hence, the embodiment of a body part modulates the way in which multisensory integration occurs around us, and, in turn, multisensory integration can be used to highlight the process of embodiment itself.

In the investigation of multisensory integration, the computation of sensory stimuli can be assessed in reference to the somatotopic map or in reference to the external, egocentric space. On one hand, the former can be achieved through visuo-tactile integration (VTI) tasks. In a VTI, the participant has to respond to a stimulus delivered to a limb, while a concurrent incoming visual stimulus is delivered at different distances. The closer to the body the visual stimulus is, the faster the reaction time (RT). Since VTI tests the area of visuo-tactile integration, which is extended by an embodied extracorporeal tool, it has been employed to assess embodiment in the animal and in humans (Iriki et al., 1996; Maravita and Iriki, 2004).

The same approach, albeit using audio-tactile stimuli, has been recently adopted to assess the extension of PPS as a proxy of tools (Canzoneri et al., 2012) and prosthesis embodiment (Canzoneri et al., 2013a). In these paradigms, an overall speeding up of RT expresses general better performance, while a variation of the shape of the RTs/distance curve is a clue of enlargement of peripersonal space (Spaccasassi et al., 2019) that may occur following tool/prosthesis embodiment.

On the other hand, the computation of sensory stimuli in reference to the external, egocentric space can be investigated through a temporal order judgment (TOJ) task (Yamamoto and Kitazawa, 2001a). In the TOJ, the participant states which one of two tactile stimuli, delivered to the upper limbs with variable asynchrony, is perceived first. When TOJ is tested with uncrossed hands, the right hand is in the right side of the environment and the judgment of which hand was stimulated first can rely only on somatosensory stimuli. When TOJ is tested with the hands crossed, the performance typically deteriorates because the tactile somatotopic spatial coordinates come in contrast with the visual external spatial coordinates conveyed by vision (Yamamoto and Kitazawa, 2001a; Schicke and Roder, 2006). The increase of RT is due to the time needed for the resolution of conflict between sensory modalities (Shore et al., 2002) or for their integration (Heed et al., 2015). Critically, the embodiment of tools (Yamamoto and Kitazawa, 2001b) and prostheses (Sato et al., 2017) is subjected to the same hand crossing effect, which has been taken as a hint to the embodiment of such bodily extensions.

In the present study, we recruited a chronic amputee volunteer and implanted intraneural and perineural multichannel electrodes on her stump to deliver sensory feedback from the prosthesis. After a period of training, she was able to perform grasps and dexterous manipulation, thanks to a closed-loop control enabled by neural feedback (Zollo et al., 2019). In this volunteer, we longitudinally investigated prosthesis embodiment, using both VTI and TOJ tasks. Experiments have been designed to assess the impact of three factors on prosthesis embodiment: (i) type of stimulation (intraneural invasive vs non-invasive), (ii) training, and (iii) type of prostheses (anthropomorphic vs more trained).

Compared to previous work, this is the first study that longitudinally investigates multiple determinants of prosthesis embodiment through proxies of body representation not derived from the rubber hand illusion or telescoping assessments, but investigating how the relation that multisensory integration has with the body impacts on reaction time. This has been done in a participant naïve for active prosthesis use and in a context of ecologic continuative use of a worn and neurally interfaced hand prosthesis.

The impact of intraneural stimulation on embodiment was investigated because invasive stimulation showed to achieve a more physiological sensory feedback (Raspopovic et al., 2014; Ciancio et al., 2016; Oddo et al., 2016; Valle et al., 2018; Zollo et al., 2019), and the valence and features of sensory feedback are recognized as enabling factors of embodiment (Blanke, 2012; D’Alonzo et al., 2019). Moreover, protocols testing multisensory integration seem to be well-suited to assess the performance of intraneural stimulation conveying information from the hand. Indeed, in monkeys implanted with intracortical array (Dadarlat et al., 2015) and in an amputee volunteer implanted with intraneural electrodes (Risso et al., 2019), multichannel invasive sensory feedback was recently shown to be optimally integrated with visual information, enhancing the precision of the estimation of position and posture of the hand. Training was investigated because it has been shown to facilitate the embodiment process (Iriki et al., 1996; Maravita et al., 2002; Farne et al., 2005) and because its impact was previously showed in healthy subjects taught to use a mechanical hand (Marini et al., 2013). Embodiment was also assessed in relation to the anthropomorphism of the prosthesis, due to a reported stronger embodiment for more human-like non-corporeal objects (Tsakiris et al., 2010).

To evaluate statistical significance of the results achieved with our participant in both experiments, they were compared with the data coming from a group of healthy controls.

## Materials and Methods

### Amputee Participant, Surgery, and Electrodes

The part of this study involving the amputee participant was conducted at Campus Bio-Medico University Hospital of Rome in accordance with the Declaration of Helsinki and following amendments, and it was approved by the local Ethics Committee and by the assigned office of the Italian Ministry of Health. The volunteer participant signed an informed consent form. She is a right-handed female, 40 years old at the time of the experiment. Almost 30 years before, she was exposed to an explosion that produced a transradial left upper limb amputation.

### Surgery and Electrodes

**FIGURE 1:**
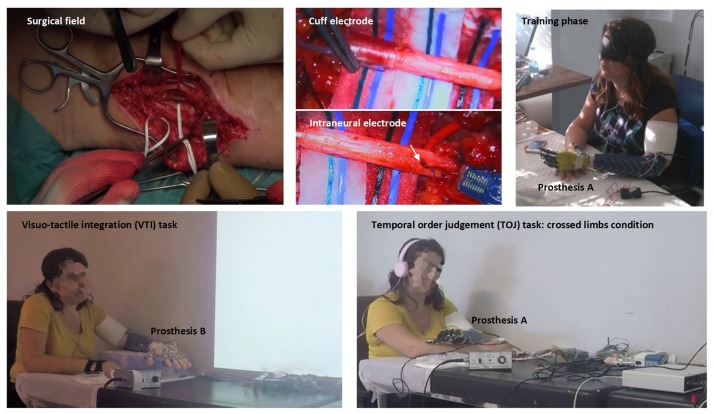
**Upper row**, from left to right: surgical field on the median aspect of the left severed arm and identification of median and ulnar nerves **(left)**; higher magnification of the perineural **(central up)** and one of the intraneural electrodes **(central down)** implanted in the nerves, participant during a blinded manipulation training session learning to exploit the neural sensory feedback to control the robotic prostheses **(right)**. **Lower row**, from left to right: the participant involved in a visuo-tactile integration (VTI) experimental session **(left)** and in a temporal order judgment (TOJ) experimental session with the arms crossed **(right)**.

The participant underwent a surgical procedure under general anesthesia where two intraneural multichannel electrodes (ds-FILEs) and one cuff electrode (Ardiem Medical Inc.) were implanted in the ulnar nerve trunk, and the same was done in the median nerve trunk (Micera et al., 2010; Di Pino et al., 2013), achieving a total of 64 intraneural plus 28 perineural channels of communication (Figure 1, upper row). Electrodes were removed 75 days after implantation to comply with the constraints of the obtained formal approval. Mild fibrotic reaction (Lotti et al., 2017) was found around the electrodes. For a full description of the surgical procedure and electrodes implanted, see Zollo et al. (2019).

### Prosthesis and Training

The volunteer subject habitually wore a cosmetic prosthesis for her everyday life, both during working hours and in social circumstances. She was naïve, though, for active prosthesis use. For the experimental tasks, she was tested with a robotic hand research prototype (prosthesis **A:** IH2 Azzurra, Prensilia s.r.l.^1^) and with a more anthropomorphic commercial device (prosthesis **B:** RoboLimb, TouchBionics s.r.l. now commercialized by Ossur^2^). Prosthesis A is an optimal robotic hand research platform, mostly open and flexible to be utilized in different lab experiments on human grip and manipulation. Prosthesis B is designed for amputee end-users to be employed in their daily living tasks at home, and during their social activities. The shape of prosthesis B was closer to the one of the human hand (e.g., the proportion of finger lengths), and its weight and size were very similar to the ones of our participant contralateral healthy hand (Table 1).

**TABLE 1:** Hand and prostheses size, weight, and shape.

	Hand weight (g)	Palm length (mm)	Palm width (mm)	Palm thickness (mm)	Palm and back shape	Fingers rest posture	I digit length (mm)	III digit length (mm)
Healthy hand	450*	100	83	34	Curved	Partly flexed	71	85
Prosthesis A	640	116	102	45	Flat	Fully extended	103	103
Prosthesis B	507	104	75	35	Curved	Partly flexed	78	87

*The weight of the healthy hand is expressed considering a percentage of 0.66% of the whole-body weight (Tözeren, 1999).*

During training, both prostheses were controlled through surface EMG sensors (Ottobock 13E200) embedded into the socket, while forces of interaction with objects were measured with force-sensing resistors (Interlink Electronics Inc.) embedded in both prostheses’ fingers and fed back through neural interfaces.

An *ad hoc* developed algorithm based on non-linear logistic regression allowed to perform power, pinch, lateral grasps, rest, open the hand, and to apply three levels of force. Every time the participant manipulation was evaluated, she performed 24 repetitions of each of the following four tasks: (A) Lateral grasp of large and small objects; (B) pick and place of large objects with a power grasp; (C) pick and place of small objects with a precision grasp; and (D) manipulation tasks featuring: pouring water from a bottle to a cup and sorting cylindric and circular-shaped objects (Zollo et al., 2019). In all the training period, the participant learned to exploit neural feedback to control both prostheses during grasps and manipulation. During the training period, the participant trained about 4 h, six times per week, while she did not use any active prosthesis in her everyday life. The improvement was monitored after the first week of training with the closed-loop control, in the middle of the training period, and at the end of the experimental study. It was assessed by means of instrumented objects and a purposely developed metrics (Zollo et al., 2019).

Due to time constraints connected with the clinical procedures, the training with prosthesis A lasted approximately 45 days, while training with prosthesis B only 20 days. Thus, prosthesis A was the less anthropomorphic and the more trained, while prosthesis B was the more anthropomorphic but the less trained.

### Experimental Design

**FIGURE 2:**
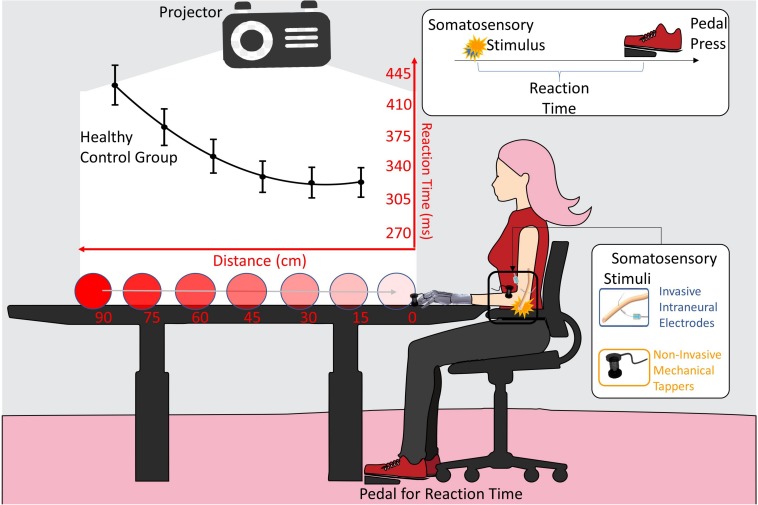
VTI experimental setup. The somatosensory stimulation was delivered either invasively (blue) through intraneural electrodes, or non-invasively (orange) through mechanical tappers, when the visual stimulus, approaching the participant, was at six possible distances (90, 75, 60, 45, 30, 15 cm). Participant had to respond to somatosensory stimuli as fast as possible. Reaction time (RT) to the somatosensory stimulus was taken with a pedal. The black line is the RTs/distance curve (quadratic fitting function) of the healthy subjects control group. Values are means (dots) ± SEM (bars).

**FIGURE 3:**
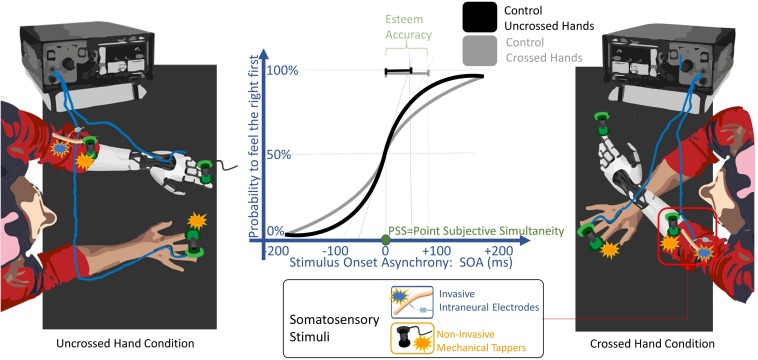
TOJ experimental setup. A pair of somatosensory stimuli were delivered non-invasively (orange) through mechanical tappers, and only in the POST_I session to the left severed limb invasively (blue) through intraneural electrodes. Stimuli were delivered, one to each limb, with a stimulus onset asynchronies (SOA) randomly assigned from 15 to 200 ms. The participant had to discern in which limb the first stimulus was delivered. The task was performed either with uncrossed and with the crossed arms. The solid lines are the probability to feel the right limb stimulated first depending on the SOA, fitted with a sigmoid function, when the task was performed by a healthy subject control group with the arms uncrossed (black) and crossed (gray). The point of subjective simultaneity (PSS) is the SOA where both limbs have the same probability to be perceived as firstly stimulated. The decrease of the slope of the curve from uncrossed to the crossed condition represents the worsening of esteem accuracy typical of the arm crossing effect. The esteem accuracy (EA) is computed through the SOA corresponding to half of the inverse of its derivate for PSS (black and gray segments for uncrossed and crossed hands, respectively); thus, the shorter the EA value is, the higher the accuracy is.

In both VTI and TOJ experiments, the participant was placed in a silent and dimly illuminated room and was acoustically shielded with white noise playing headphones to cover the noise produced by the tappers (Figure 1, lower row; Figures 2, 3). Visual and tactile stimuli were presented by the open-source “OpenSesame” software v.3.1 (Mathôt et al., 2012).

**FIGURE 4:**
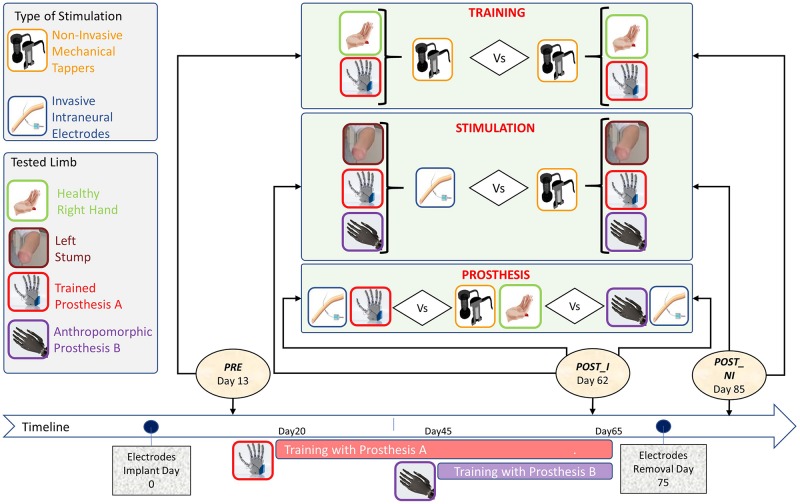
Timeline and protocol design for the three tested factors: (i) Training, (ii) Stimulation, and (iii) Prosthesis, with the types of stimulation (left-up panel) and tested limbs/prostheses (left-center panel).

In order to assess the achievement of proficiency due to the training, the participant was tested at three different time points (Figure 4):

• (*PRE*) Pre-training, non-invasive stimulation: baseline measure, taken when the electrodes were already implanted but the subject had not yet started the training with the prosthesis. Stimulation was delivered to the stump through mechanical tappers.
• (*POST_I*) Post-training, invasive stimulation: 50 days after *PRE* and after 30 days of training with the prostheses. Somatosensory stimulation was delivered to the severed limb through intraneural electrodes.
• (*POST_NI*) Post-training, non-invasive stimulation: after electrodes removal. Stimulation was delivered to the severed limb through mechanical tappers.

Visuo-somatic multisensory integration tasks were performed to assess the impact of the type of stimulation, training, and anthropomorphism on prosthesis embodiment (Figure 4).

(i) To test the impact of invasive stimulation on the achieved embodiment, the data from two sessions were compared: *POST_NI* (post_non invasive) collected after electrodes removal was compared with *POST_I* (post_invasive) performed while the participant still had the intraneural electrodes implanted. In both sessions, the healthy limb was stimulated non-invasively. In *POST_I* the stimulation was delivered intraneurally to the affected left limb. For a comprehensive description of the features and location of referred sensation, please see Zollo et al. (2019).
(ii) To investigate the impact of training on embodiment of neurally interfaced prosthesis, the participant, who was naïve for active prosthesis use, was tested non-invasively before (*PRE*), and after training (*POST_NI*).
(iii) To investigate embodiment in relation to the anthropomorphism of the prosthesis, in the *POST_I*, the participant was tested while wearing the two different prosthetic devices (Prosthesis **A** and Prosthesis **B**).

Two different groups of healthy subjects were enrolled as controls for the two experiments. Thirty-six participants (13 males, age = 24.11 years, SD = 4.15, range: 19–42) performed the VTI task and 11 participants (6 males, age = 26.36 years, SD = 3.34, range: 23–32) performed the TOJ. This research was approved by the local Ethics Committee of the Department of Psychology, University of Milano-Bicocca.

### Somatosensory Stimulation

As mentioned in the previous paragraph, experiments were designed to deliver sensory stimuli on the fingertips of both hands’ index finger and were of two types:

(i) Non-invasive tactile stimuli, administered by means of solenoid tappers (magnetic rod diameter: 4 mm) controlled by an *ad hoc* built relay box (Tactile Box, EMS, Bologna, Italy). Healthy participants, used as control groups, were always stimulated non-invasively. Non-invasive stimulation was also always used for stimulating amputee healthy right upper limb, as well as in *PRE* and *POST_NI* to stimulate her severed left limb. This was achieved by placing the tapper right upon the area of the skin that elicited, in the volunteer, the feeling of being touched on her phantom left index fingertip. The area where the fingers were reported to be was identified with two methods: firstly, by asking the subject where she felt a referred sensation of any digit, and secondly, by touching the skin of the stump until she referred to be touched on that finger. For all the fingers, the maps identified by the two methods were coherent. The skin area marked as digit II was employed as stimulation point. An inactive tapper was placed upon the prosthesis index finger to emulate the one placed on the healthy hand and to reinforce the perception in the volunteer that the stimulation was coming from the index finger on both sides.
(ii) Intraneural invasive stimulation was exploited in *POST_I* to stimulate the amputee participant severed left limb, employing parameters that induced a sensation that she referred to be closer to the one evoked by the mechanical tapper, in terms of intensity, modality, and referred territory. Indeed, after electrode implantation, a whole-contact psychophysical sensory mapping was performed to establish the match between stimulated contact, modality amplitude, and referred territory of the evoked sensation. Moreover, sensory mapping has been tested and retested day after day before *PRE* to have an estimation of the day-by-day reliability of the evoked sensations. The neural electrodes elicited sensations that the participant referred to 13 different locations of the anterior and the posterior parts of the phantom hand. The contacts that were used for the real-time closed-loop control of the prosthesis were chosen because they: (i) did not evoke muscle twitch at the beginning of the test period and (ii) changed over time the induced sensation from eliciting the sensation of movement to the sense of touch. In both tasks, invasive stimulation was delivered in the form of square pulse stimuli to the channel number 12 of the intraneural electrode (ds-FILE) positioned proximally within the median nerve (stimulus intensity 300 μA, duration 200 μs) (Intraneural Stimulator: Grass S88X, Astro-Med Inc., West Warwick, RI, United States). Stimulation parameters and channel were selected on the basis of the sensations reported by the participant in multiple assessment tests. For a comprehensive description of the features, stability, and location of referred sensations, please see Zollo et al. (2019).

### VTI Task

In this experiment, a VTI task was carried out to assess the extent of the peripersonal space (PPS) in different conditions and at different time points.

#### Experimental Setup and Procedure

The participant was seated beside the wall with her tested upper limb resting on a table in a prone position (Figure 1, lower row; Figure 2). A PC-driven digital projector was set at a distance suited for projecting a video that covered a 100 × 75 cm surface on the wall. The video showed a visual stimulus approaching the participant. Visual stimuli consisted of looming images on a white background covering a distance of 1 m on the bottom side of the projecting area and traveling at a constant speed of 66 cm/s.

On all testing sessions, the space of VTI was tested for the participant’s right healthy limb, the severed left limb wearing prosthesis **A** and prosthesis **B** (in PRE session, prosthesis **B** was not available), and the bare stump. The participant’s elbow was always kept at 42 cm from the limit of the projecting area. This resulted in a distance from the projecting area of 30 cm from the endpoint of the stump, while in all the other conditions, the extremity of the limb or the prosthesis was in contact with the limit of the projecting area.

Along with the visual stimulus, in 85% of the trials, a tactile stimulus was randomly delivered when the visual approaching stimulus was at one of six distance points (15, 30, 45, 60, 75, 90 cm) from the end of the projecting area. The participant was asked to press a pedal as soon as she perceived the tactile stimulus. The remaining 15% of the trials were “catch trials,” where no tactile stimulus was administered and no response was expected.

The task lasted approximately 8 min, consisting in a total of 168 trials (24 repetitions per distance points and 24 catch trials) and 800 ms of inter-trial interval. A small training phase with 15 stimuli presentation preceded the experimental task.

#### Data Analysis

Data were analyzed by addressing the three experimental questions defined in the section “Introduction.” Analysis was implemented in order to weigh different factors, while minimizing the total number of comparisons. The dependent variable which was taken into consideration was the RT in response to the tactile stimulus (Zangrandi et al., 2019). The factor *Distance* was computed as a continuous variable.

• STIMULATION. In order to assess the relative weight of the stimulation interface (*non-invasive* vs *intraneural*), we implemented a *linear mixed model* (*LMM*). The model was analyzed with an analysis of variance (ANOVA) with Satterthwaite approximation for degrees of freedom. We included as predictors the continuous factor *Distance* and the dichotomous factor *Stimulation* (*intraneural* vs *non-invasive*). The number of the trial was added as a random effect variable. The two levels of factor Stimulation have been implemented by pooling together all the conditions exploiting intraneural interface at *POST_I* (prosthesis **A** + prosthesis **B** + bare stump: level *intraneural)* tested against the same conditions recorded at *POST_NI* (level *non-invasive*). To maintain homogeneity of samples, data from *PRE* were not included because they lack prosthesis **B**. Before being available to perform the non-invasive stimulation session, the participant had to go through all the activities linked with presurgical exams, surgery for electrode removal, and recovery after the surgery. Thus, *POST_NI* session was performed 22 days after the *POST_I* session. The time spent training with the prostheses between *POST_I* and *POST_NI* was marginal because the participant was involved in perioperative procedures; thus, the impact of any additional training between the two sessions should be considered negligible.
• TRAINING. In order to assess the relative weight of the training to control the prosthesis, we used the same approach of stimulation analysis. We employed a LMM, analyzed with an ANOVA with Satterthwaite approximation for degrees of freedom. The number of the trial was added as a random effect variable. Predictors were the continuous variable *Distance* and two dichotomous variables: *Hand* (prosthesis **A** vs healthy limb) and *Time* (*PRE* vs *POST_NI*). We have chosen these time points to avoid introducing noise in the data due to different types of stimulation (at *POST_I* stimulation was delivered intraneurally). Additionally, in order to highlight the effects of training on the performance of the task, we conducted two independent ANOVA with the predictors *Distance* and *Time* (*PRE* vs *POST_NI*).
• PROSTHESIS. In order to assess the relative weight of the employed prostheses, and whether they were embodied, we again employed a LMM, analyzed with an ANOVA with Satterthwaite approximation for degrees of freedom. The number of the trial was added as a random effect variable. Predictors were *Distance* and *Hand* (three levels: prosthesis **A**, prosthesis **B**, and healthy hand). The three levels of factor *Hand* have been implemented with data recorded at *POST_I*. *POST_I* has been chosen because it was the only time point when prosthesis feedback could have been done through intraneural stimulation, and because the proficiency with the prosthesis was already achieved. Additionally, we ran an analysis to evaluate the performance in the spatial transition from peripersonal to extrapersonal space. We selected the distances of 15 (near) and 45 (far) cm. When the participant was tested on the stump condition, thus not wearing any kind of prosthesis, the tip of the stump was 30 cm away of the tip of the prosthesis (e.g., closer to the trunk). By doing so, the same distances resulted in a 45 (near) and 75 (far) cm away, although the physical positions of both the visual stimulus and stump were exactly the same in both conditions. Thus, we adopted a LMM, analyzed with an ANOVA with Satterthwaite approximation for degrees of freedom including the number of trials as a random effect variable. The predictors were the *Distance* (near vs far) and *Hand* (prosthesis **A** vs stump).

### TOJ Task

In this experiment, a TOJ of two stimuli delivered on the upper limb was carried out to investigate the participant’s body awareness in different conditions and at different time points.

#### Experimental Setup and Procedure

The participant was seated in front of a table, with both her upper limbs lying on its surface in a prone position (Figure 1, lower row; Figure 3). Two tactile stimuli were delivered rapidly, one to each limb, with one of the following randomly assigned stimulus onset asynchronies (SOA): -200, -90, -55, -30, -15, 15, 30, 55, 90, 200 ms. Negative intervals indicate that the right limb was stimulated before the left limb and *vice versa*.

Each trial started with a visual cue (100 ms red LED light) which was followed, after 300 ms, by two tactile stimuli, delivered one to each limb. Before the experiment started, two colored stickers were applied to the participant’s arms (the association between color and arms varied across the testing conditions) and they were used as a code to indicate the stimulated limb without referring to laterality tags (left/right).

Each experimental condition was tested with eight experimental blocks, four while the subject’s hands were uncrossed (with a gap of 40 cm between her hands) and the other four when her hands were crossed. In the crossed conditions, in half of these blocks, the right limb was kept over the left limb and in the other half, the left limb was kept over the right limb. In four experimental blocks, the participant was asked to verbally report whether the first of the two stimuli was administered on the right or the left limb, while in the other four, on the contrary, she had to report where the second stimulus occurred.

Testing each condition, consisting in a total of 200 trials (20 repetitions per SOA) for the uncrossed limbs and the same amount of trials for the crossed limbs, lasted approximately 35 min. In *POST_I*, the task was repeated twice, using either prosthesis **A** or prosthesis **B.**

#### Data Analysis

The order judgment of the subject in each condition was plotted with the different “*stimulus onset asynchrony (SOA)*” as independent variable (x-axis) and “*the probability to judge the right limb as the one firstly stimulated*” as the dependent variable (y-axis). Then, data distribution was fitted with a psychophysics sigmoid function:

$$\text{P}\,\left(\mathrm{SOA},\mathrm{PSS},\mathrm{EA}\right)=\frac{1}{1+\exp\left(-\frac{\text{SOA}-\text{PSS}}{0.5\times \text{EA}}\right)}$$

where the two parameters PSS and EA represent:

Point of subjective simultaneity (PSS):

$$\text{PSS}={\text{SoA}|}_{P_{0.5}}$$

This is the SOA value on the curve where the first stimulus had the same probability (*p* = 0.5) to be felt on the right and on the left limb. It testifies the laterality stimulation bias measured in milliseconds.

Esteem accuracy (EA):

$$\text{EA}={\left({2\times \frac{d\,\text{P}}{d\,\text{SOA}}|}_{\mathrm{SOA}=\mathrm{PSS}}\right)}^{-1}$$

This is the SOA needed for the line tangent to the curve at (*p* = 0.5) to reach the value *p* = 1. It is the inverse of the slope of the curve multiplied by 0.5. The shorter it is, the more accurate the esteem.

Fitting TOJ data with the previous function gives back a value of PSS and EA for each tested condition. To give statistic relevance to those outputs, they were compared with the ones obtained by the control group of healthy subjects performing the same task, by means of Crawford *t*-tests.

The Crawford t-test, instead of comparing the performance with that of a large population with a normal distribution, matches the participant’s score against a relatively small control group (i.e., frequently *N* < 10 and typically up to 30). The control group must have done exactly the same task of the single case participant, then a Student-*t* distribution was adopted for matching the participant’s performance. Under simulations, the method proved to reliably keep under control the alpha error probability to the nominal value of.05 (Crawford and Garthwaite, 2007; Crawford et al., 2010). We also report the effect size (Z-CC), an index analogous to the Cohen’s d and the limit of the credibility intervals (CI) of the effect size.

• STIMULATION: Pooling together the bare stump, prosthesis **A** and prosthesis **B**, two different pair of stimulations (in *POST_I*: right limb stimulated non-invasively and left limb stimulated invasively vs in *POST_NI* both limbs stimulated non-invasively) were compared through their PSS in the uncrossed condition. The uncrossed condition is the standard situation of the TOJ task, without conflicting information about laterality, thus results in the highest accuracy. Therefore, this is the best condition to evaluate any laterality temporal bias which may be only due to the type of stimulation.
• TRAINING: In *PRE* session, the participant was extremely inaccurate and variable in performing the crossed condition, so data have not been analyzed.
• PROSTHESIS: To assess embodiment of prostheses with intraneural sensory feedback, we evaluated in *POST_I* the worsening of Esteem Accuracy due to hand crossing, while the participant was wearing prosthesis **A** or prosthesis **B**.

## Results

### Stimulation

**FIGURE 5:**
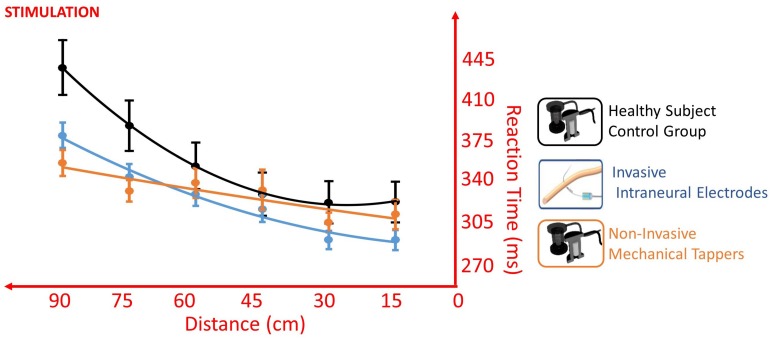
Different RT/Distance curves (quadratic fitting function) in the VTI experiment of Invasive Intraneural Stimulation (blue) and Non-Invasive Stimulation (orange) compared to the pattern of the control group (black). Values are means (dots) ± SEM (bars). The absence of interaction between Intraneural and Control [*F*(1,5033) = 3.3954, *p* = 0.066], compared to the presence of interaction between Non-Invasive and Control [*F*(1,5077) = 28.379, *p* < 0.001], supports a facilitation of prosthesis embodiment when this was tested with intraneural stimulation.

The VTI experiment first replicated the well-known reduction of RT when the somatosensory stimulus was delivered while the visual stimulus was closer to the upper limb extremity, with a main effect of *Distance* [*F*(1,766) = 47.758, *p* < 0.001]. Critically, the participant accomplished the task differently according to the type of somatosensory stimulation, as shown by the main effect of *Stimulation* [*F*(1,760) = 5.842, *p* = 0.016]. Moreover, the type of stimulation affected the pattern of the RT/distance curve, as shown by the interaction *Distance* × *Stimulation* [*F*(1,766) = 5.544, *p* = 0.019] (Figure 5).

When the participant was stimulated non-invasively, the presence of clearly significant *Distance* × *Group* interaction (*amputee stimulated non-invasively* vs *Control*) [*F*(1,5077) = 28.379, *p* < 0.001] suggests that the participant behaved differently from the control healthy group. The same remarkable difference was not observed when the participant was stimulated invasively (*Distance* × *Group* – *amputee stimulated invasively* vs *Control*: [*F*(1,5033) = 3.3954, *p* = 0.066], showing a behavior similar to that of the control group (Figure 5). Having a similar RT/Distance pattern than healthy control is in favor of facilitation of prosthesis embodiment when this was tested with intraneural stimulation.

In the TOJ with uncrossed limbs, when the right limb was stimulated non-invasively and the left invasively, there was a significant right laterality bias, so that the participant perceived intraneural left stimulation with about 30 ms delay compared to the healthy limb [point of subjective simultaneity (PSS) = + 28.9, CI: (+14.8 ms, +42.5 ms)]. PSS was significantly different than the one of the control group only when the left limb was stimulated invasively [non-invasive/intraneural vs control: *t*(10) = 2.582, *p* = 0.027, Z-CC = 2.69 CI (1.37, 3.98); noninvasive/non-invasive vs control: *t*(10) = 0.946, *p* = 0.336)].

**FIGURE 6:**
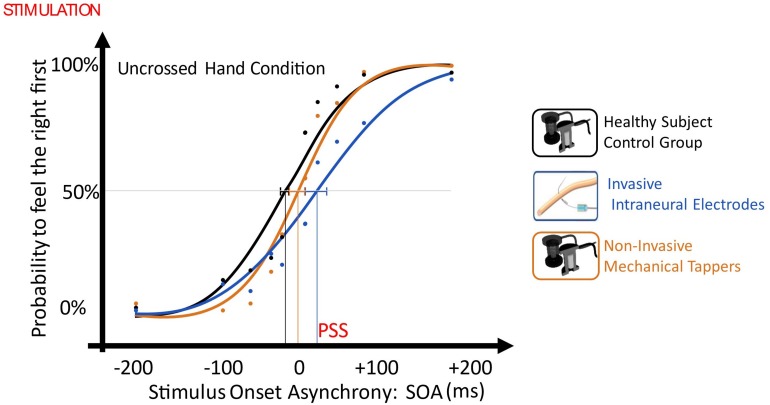
Plot of uncrossed hand order judgment with the different SOAs as independent variable (x-axis, ms) and the probability to judge the right limb as the one stimulated first, as the dependent variable (y-axis). Then, data distribution was fitted with a sigmoid function, and the SOA value in the curve where the first stimulus had the same probability (*p* = 0.5) to be felt on right and on the left limb was defined as Point of Subjective Simultaneity (PSS) and it testifies the laterality stimulation bias. The dashed lines represent PSS 95% confidence intervals. Blue: Invasive vs Non-Invasive; Orange: Non-invasive vs Non-Invasive; Black: Control.

When the left limb was stimulated intraneurally and the right limb non-invasively, the performance of the task was not worse than when both limbs were stimulated non-invasively, despite the asymmetric stimulation. Indeed, both conditions had esteem accuracy (EA) not different than the one of the control group (non-invasive/intraneural vs control: *t* = 1.011, *p* = 0.336; non-invasive/non-invasive vs control: *t* = -0.383, *p* = 0.71) (Figure 6).

### Training

**FIGURE 7:**
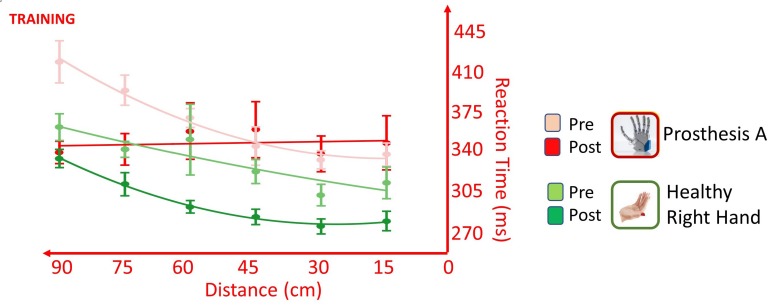
Effect of training on RT/Distance curves (quadratic fitting function) in the VTI experiment, when the participant was tested with the healthy right limb (green) and with the more trained prosthesis (red) in the PRE session (lighter colors) and in the POST_NI session (darker colors). Somatosensory stimulation was always delivered non-invasively. Values are means (dots) ± SEM (bars). The healthy hand underwent a general speeding of RTs for all the tested distances yet maintaining a similar RT/Distance pattern, while the prosthesis changed the pattern of the RT/distance with a decrease of RTs in far space. This suggests an extension of the PPS induced by the embodiment of the prosthesis.

The training changed the way the participant accomplishes the task, depending on the hand tested; this was suggested by the presence of significant *Hand* × *Time* [*F*(1,537) = 8.166, *p* = 0.004], *Distance* × *Time* [*F*(1,549) = 5.965, *p* = 0.015] and *Distance* × *Hand* × *Time* [*F*(1,550) = 5.990, *p* = 0.015] interactions. The training induced in the healthy limb a general speeding up of RTs for all distances, as suggested by the presence of main effect of *Time* [*F*(1,271) = 5.523, *p* = 0.019], but it did not change the pattern of the RT/distance; absence of *Distance* × *Time* interaction [*F*(1,277) = 0.011, *p* = 0.916]. Conversely, testing the prosthesis, the training changed the pattern of the RT/distance curve as shown by the presence of interaction *Distance* × *Time* [*F*(1,277) = 10.764, *p* = 0.001], besides the main effects of *Distance* [*F*(1,277) = 8.780, *p* = 0.003] and *Time* [*F*(1,277) = 4.392, *p* = 0.037]. Change of pattern, with a decrease of RTs in far space, going from 60 to 90 cm, suggested an extension of the PPS and it is in favor of a positive effect of training on the embodiment of the prosthesis (Figure 7).

The TOJ task did not give additional information on the effect of training since, in the PRE, the participant was extremely inaccurate and variable in performing the crossed hand condition (EA: uncrossed SOA = 70 ± 15 ms vs crossed SOA = 220 ± 140 ms) and data have not been analyzed.

### Prosthesis

**FIGURE 8:**
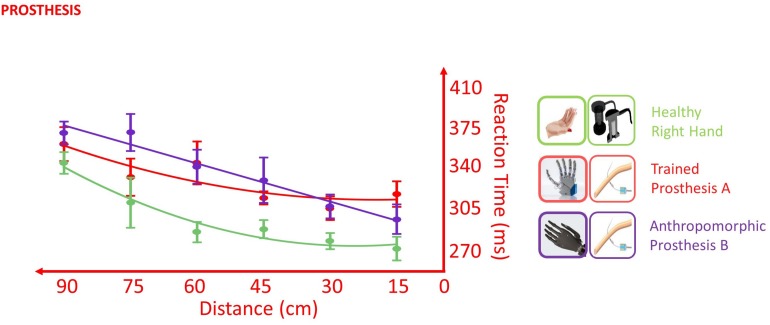
Effect of different prostheses on RT/Distance curves (quadratic fitting function) in the VTI experiment. Values are means (dots) ± SEM (bars). In the POST_I session when the participant was tested with both the prostheses, she was slower for all the tested distances than when she was tested with the right healthy limb (green); however, the RT/Distance pattern was not different (Distance × Hand interaction *p* = 0.309), which is in favor of an embodiment of both prostheses.

In the VTI task, the healthy limb was in general more rapid than both the tested prostheses, as suggested by the presence of a main effect of *Hand* [*F*(2,338) = 4.136, *p* = 0.017]. The direct comparison between the levels of the factor *Hand* showed that the healthy limb was different from both the prosthesis **A** (*p* < 0.001) and the prosthesis **B** (*p* < 0.001), while prosthesis **A** did not differ from prosthesis **B** (*p* = 0.428). Despite the different average RT, the two prostheses and the healthy limb did not show a statistically different pattern of response across the distances (*Distance* × *Hand* interaction: *F*(2,351) = 1.180, *p* = 0.309), suggesting that the stimuli approaching the prosthesis were processed similarly to those approaching the healthy limb. This is in favor of an embodiment of both prostheses (Figure 8).

**FIGURE 9:**
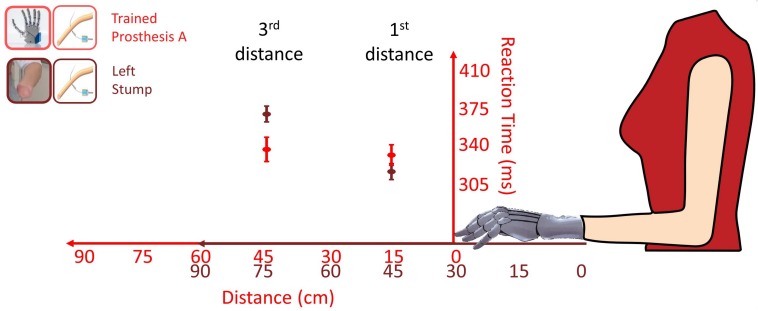
Comparison of RTs (mean ± SEM) for the first and third distance tested with the more trained prosthesis (red) and with the bare stump (brown) in the POST_I session stimulating invasively through the intraneural interface. The red values correspond to the distances of the visual stimulus from the Prosthesis A; the brown values correspond to the distances of the visual stimulus from the bare stump. The passage between peripersonal to extrapersonal space, which seems to be between those distances, appears to shifted forward when the participant was tested with the prosthesis.

Moreover, VTI gave an additional cue of prosthesis embodiment. The first and third distances, i.e., 15 and 45 cm from the prosthesis respectively corresponded to 45 and 75 cm from the stump, because the tip of the stump was 30 cm shorter than the tip of the prosthesis. Considering the bare stump, the shift from PPS to extrapersonal space would likely fall within a 45–75 cm range (Serino et al., 2015), so that when the stump was tested, there was an important decrease of RTs from the third distance (RT = 368) to first distance (RT = 314 ms). An embodied prosthesis would shift the boundary of peripersonal space, so that both first and third distances would fall within the PPS, because they correspond to 15 and 45 cm from the prosthesis (Figure 9). Indeed, when prostheses were tested, there was almost no RT difference (from 3rd = 335 to 1st = 330 ms). Prosthesis embodiment was statistically confirmed by the presence of a significant *Distance* (1st vs 3rd) × *Hand (stump vs prosthesis)* interaction [*F*(1,256.92) = 8.3077, *p* = 0.004].

So far, the VTI task showed that both prostheses tested with intraneural stimulation behaved as the healthy limb, thus suggesting their embodiment, while the TOJ gave contrasting results.

**FIGURE 10:**
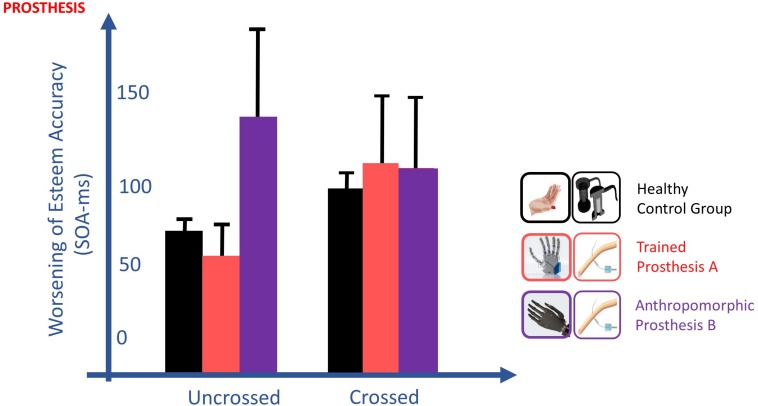
Changes of esteem accuracy (EA) between uncrossed and crossed-arm TOJ in the POST_I session. EA is computed through the SOA corresponding to half of the inverse of its derivate for PSS; thus, the shorter the SOA, the better the accuracy is. The worsening of EA going from the uncrossed to crossed hands, typical of healthy subjects (black), was present only for the more-trained prosthesis (red) and absent for the more anthropomorphic (violet) prosthesis. TOJ crossing hand effect is in favor of the embodiment of only the more trained prosthesis.

In the TOJ, the worsening of esteem accuracy going from the uncrossed to crossed hands, typical of healthy subjects (EA control group: 66.3 vs 97.2 ms), was present only for the less-anthropomorphic more-trained prosthesis **A** (EA: 55.9 vs 114.1 ms). Indeed, esteem accuracy with this prosthesis was not significantly different with the ones of the healthy subject control group, both in crossed [*t*(10) = 0.480, *p* = 0.642] and uncrossed hand [*t*(10) = 0.432, *p* = 0.675] conditions. By contrast, the crossed/uncrossed difference was absent for the more-anthropomorphic less-trained prosthesis **B** (EA: 121.7 vs 113.5 ms), where esteem accuracy was significantly different from controls in the uncrossed hand condition [*t*(10) = 2.298, *p* = 0.044, Z-CC = 2.39 CI (1.19, 3.57)] and not in the crossed hand condition [*t*(10) = 0.463, *p* = 0.653] (Figure 10).

## Discussion

This study was designed to investigate the embodiment achieved in a context of ecologic continuative use of a worn and neurally interfaced hand prosthesis. Embodiment was favored by the closure of the sensorimotor control loop of the prosthesis, enabled by a more natural and rich sensory feedback delivered through multichannel neural interface.

It has peculiar features compared to the previous studies approaching prosthesis embodiment. We had the opportunity to test our subject longitudinally, before and after a one-month period when she achieved proficiency in controlling the prosthesis. Moreover, the studied subject had a stable chronic amputation; thus, at the time of the study, she was not going through any spontaneous recovery plasticity, and she had not used active prostheses before the study, allowing us to have a clean measure of her baseline prosthesis embodiment at the beginning of the experiment.

Amputee subjects carrying intraneural electrodes implantation to receive prosthesis sensory feedback are still rare; thus, single-case studies are worth running to gather clues on the embodiment of neurally interfaced prostheses. To minimize possible biases linked with studying single cases, we decided not to rely on subjective and explicit statements, but to employ two implicit and objective paradigms, to test-retest the subject, and to statistically compare the results from the volunteer with those from a healthy subject control group.

The paradigms investigated prosthesis embodiment through the study of multisensory integration and spatial remapping; they were developed in healthy subjects (Yamamoto and Kitazawa, 2001a; Gray and Tan, 2002; Shore et al., 2002) and demonstrated to be sensible to the embodiment of tools (Yamamoto and Kitazawa, 2001b; Canzoneri et al., 2013b) and were already validated in amputees (Canzoneri et al., 2013a; Sato et al., 2017).

During the period of training, the participant used both prostheses, always receiving a rich tactile feedback related to manipulation activities made with the prosthesis through intraneural and perineural multichannel electrodes. However, when the participant was tested in the multisensory integration tasks, somatosensory stimuli were delivered in different sessions, either with non-invasive mechanical tappers or with invasive intraneural stimulation to highlight possible advantages of the latter on embodiment.

Intraneural stimulation did not speed VTI RT, and when TOJ was tested by stimulating the right limb non-invasively and the left limb invasively, there was a laterality bias toward the healthy right limb. Indeed, intraneural stimulation had to be delivered about 30 ms before the contralateral to have the same probability to be felt as the first. Since electrodes are implanted in the median nerve of the arm and since intraneural stimulation does not need mechanoreceptor transduction, the longer duration of the tapper stimulation (40 ms vs 200 μs) has likely hidden the perception of the intraneural stimulation.

Being the electrode implanted only in the left limb, we could not test TOJ performed with both sides stimulated intraneurally and demonstrate any improvement of performance due to intraneural stimulation. However, we could show that when stimulation was delivered non-invasively on the right and invasively on the left limb, TOJ performance did not become worse, despite the asymmetry of stimulation.

Even more importantly, VTI showed that with intraneural stimulation the RT/Distance pattern of the amputee wearing the prosthesis was more similar to the one of the healthy subject control group. This normalization of the relation between multisensory integration and distance from the body is in favor of a selective advantage of intraneural stimulation on prosthesis embodiment.

To the best of our knowledge, this is the first study that demonstrated in an amputee that training the control of the prosthesis favors the process of embodiment by testing-retesting the participant before and after the training period. To avoid any bias due to different stimulation, the effect of training was always tested with the same stimulation, i.e., non-invasively.

During the month of training, the participant was often involved in performing skilled bimanual tasks; thus, both limbs were trained. When the effect of training on the PPS expansion was assessed through the VTI task, we found a differential effect depending on the tested limb. The healthy limb showed an overall increase of performance, boosting up the effect of incoming visual stimuli on touch detection, thus decreasing RTs at all distances of the visual approaching stimulus. Notably, the affected limb showed a modification of the RT/Distance pattern, with a clear extension of the PPS, which is a strong clue of extracorporeal device embodiment (Maravita and Iriki, 2004). From the TOJ task, we could not gather additional information on the effect of training because the esteem accuracy in the crossed hand condition was extremely low and variable, probably due to the rarity of exploiting the stump to act and explore the contralateral side of the space.

In the VTI, prostheses recalibrated the PPS around the subject, as shown by the shift of the PPS/Extrapersonal boundary. Indeed, when the subject was tested with the bare stump, the boundary distanced between 30 and 60 cm from the stump, and when the subject was tested wearing the prosthesis, the boundary distanced more than 60 cm. This was in line with the previously demonstrated partial recovery of PPS shrank by the amputation (Canzoneri et al., 2013a). Moreover, the participant had a similar pattern of response across distances for her right healthy limb and both prostheses, suggesting that these were similarly able to determine the extension of VTI in the PPS. Conversely, only wearing the more trained, despite less anthropomorphic, prosthesis, the participant experienced the worsening of the TOJ performance following arm crossing, comparably to the control group.

Recently, it has been shown that even prostheses unable to give any sensory feedback were able to induce the crossing hand effect, typical of healthy limbs (Sato et al., 2017). It is worth noting that in that study, the three amputees were tested with their own, daily used (thus hypertrained) prosthesis. This suggests that using the prosthesis to act in external space provides enough clues to allow the remapping of its cortical representation, inducing the relocation of somatosensory stimuli in the contralateral side, thus explaining the detrimental effect on TOJ on arm crossing.

Notably, previous studies that reported the hand crossing effect on TOJ, always stimulated the tip of the hand, drumstick, or prosthesis, which could be relocated in the contralateral space quite far from the midsagittal plane in the limb/tool crossing. By contrast, intraneural stimulation was delivered to our participant through electrodes placed on the median portion of the arm, which even in the crossed condition remained in the same side of the space, as if electrodes were uncrossed. Thus, in our case, we had a dissociation between the side where the stimulation was physically delivered and the side where it was referred. Performance worsening occurred with uncrossed real stimulation which was perceived as crossed only because of the sensation remapping, emphasizing the link between prosthesis embodiment and hand crossing effect.

Both VTI and TOJ crossing hand effect are based upon multisensory integration. Somatosensory feedbacks, such as touch, proprioception, and the efference copy are coded in an egocentric reference frame, where they are compared to “where I am.” Conversely, environmental feedbacks, such as sight and hearing, are coded in an allocentric reference frame, i.e., where the information is compared to the rest of the environment. To integrate information that does not share the same reference frame, a process of remapping is needed.

In sensory remapping, a sensory modality is not just recoded in the frame of another modality. Instead, it is recoded in a new space that mixes the spaces of the two modalities using weights in line with a policy based on Bayesian integration (Miyazaki et al., 2006; Heed et al., 2015) such as Kalman filter-like noise optimization of sensory fusion (Deneve et al., 2007). However, for the sake of simplicity, we will treat sensory remapping as a simple sequential transformation in the following discussion.

The knowledge of where the environment is in relation to the hand and the awareness of what the hand movement will affect are both needed to achieve an effective hand-environment interaction. Thus, a close sensorimotor control loop involves a double transformation: along the afferent branch, environmental information must be re-referenced in a bodily frame, while in the efferent branch, the knowledge of the body coordinates must be re-referenced in the allocentric frame of the environment.

In VTI and related acoustic-tactile integration tasks, environmental inputs collected by sight or hearing assume different valence, and are able to enhance the effect of touch, depending on where they are in respect to the body (Serino, 2019). This process subtends a re-referencing of environmental information in a bodily frame; thus, VTI tests the remapping typical of the afferent branch.

PPS is the space around the body where external events are considered more relevant. It has been suggested that we have a motor-based PPS that has the higher possibility to be directly acted upon by the body, and a defense-based PPS where external stimuli may be more effective upon the body (Clery et al., 2015b; de Vignemont and Iannetti, 2015).

In our VTI task, we tested fast approaching stimuli. Looming stimuli are potentially more dangerous than static, so that they induce a protection response with enhanced tactile sensitivity in their predicted time and site of impact (Gray and Tan, 2002; Clery et al., 2015a). It’s likely that we tested the extension of a safe margin around the body, since training was less relevant than sensory feedback in determining VTI outcome.

In the uncrossed hand condition of the TOJ task, egocentric and allocentric reference frames are concordant and somatosensory stimuli are analyzed in their original bodily referenced frame. On the contrary, with crossed hands, the delivered somatosensory stimuli assume a different value depending on where they occur in the egocentric space; thus, the judgment subtends a compulsory remapping of the location of tactile stimuli in external world coordinates (Schicke and Roder, 2006). Accordingly, we think that the TOJ hand crossing effect tests the remapping typical of the efferent branch, which is more aimed to action.

Despite TOJ being a tactile task, several cues are in favor of a motor-based origin of the transformation it tests, because external spatial coordinates allow the movement toward the tactile event. Indeed, TOJ is modulated by hand movements, which are able to compress time interval (Tomassini et al., 2014). The crossing effect is also present behind the body where the space can be only coded by movement (Gillmeister and Forster, 2012), and may be due to the efference copy since it is present when the hands are uncrossed but a crossing movement is planned (Hermosillo et al., 2011). Moreover, the crossing effect sticks with the part where the motor operational ability is focused, while the position of the rest of the arm or of the tool is irrelevant, as showed by the absence of the effect when double crossing with drumsticks (Yamamoto and Kitazawa, 2001b) and its presence with L-shape sticks (Yamamoto et al., 2005).

The participant we tested could control both prostheses with an *ad hoc* developed EMG control and received tactile feedback from the prostheses related to dexterous manipulation and slippage through invasive nerve stimulation (Zollo et al., 2019). Hence, the control loop of both prostheses benefited of invasive feedback, and this may explain why both prostheses were embodied accordingly to the VTI. Both prostheses behaved as her right real hand because they were both able to trigger the remapping of visual stimuli into bodily coordinates.

The time our volunteer spent in training skilled manipulation with each of two prostheses was very different (45 days for prosthesis **A** vs 20 days for prosthesis **B**), and this may be the reason of a different embodiment of the two prostheses according to the TOJ. Only the more trained prosthesis, despite being less anthropomorphic, was able to trigger the bodily-into-environment remapping and to induce the hand crossing effect.

Training is needed by the plastic processes at the base of remapping and neurally interfaced hand prostheses have shown a strong ability to foster such plasticity. This has been widely demonstrated in primary sensorimotor cortices (Rossini et al., 2010) and in their interplay (Ferreri et al., 2014; Di Pino et al., 2012), but it failed to be shown in the fronto-parietal network (Mioli et al., 2018). In targeted muscle and sensory reinnervated patients, which benefit of high effective bidirectional interface with the prosthesis, M1 and S1 activity and connectivity were almost normal, but the interplay with the frontal and parietal areas was highly impaired (Serino et al., 2017). Is the induction of plasticity on that network still beyond the ability of highly interacting prostheses? The present study offers a behavioral demonstration of plasticity of the frontoparietal network induced by neurally interfaced prostheses.

Indeed, TOJ has been ascribed to the activity of parietal and prefrontal cortices and the crossing hand effect to their combination with multisensory perisylvian cortices coding the representation of motion (Takahashi et al., 2013). The multisensory integration at the base of VTI has been widely ascribed to fronto-parietal interplay (di Pellegrino and Ladavas, 2015) and TMS entrainment and disruption studies highlighted the importance of posterior parietal cortex in frame remapping (Bolognini and Maravita, 2007; Konen and Haggard, 2014; Ruzzoli and Soto-Faraco, 2014).

In monkey, two partly separated networks with bimodal visuotactile neurons are responsible for multisensory integration in the PPS. The VIP-F4 is more involved in coding a defense PPS around the vulnerable parts, especially hand and face (Graziano and Cooke, 2006) and is sensible to emotional and social aspects (Clery et al., 2015b), while the areas7b and AIP-F5, which are in charge of the visuomotor transformation needed for grasping objects in the environment (Rizzolatti and Luppino, 2001), code the motor PPS. We may speculate that prosthesis embodiment revealed by VTI relies more on the former and has been achieved in our subject with both prostheses, while embodiment revealed by TOJ relies more on the latter, and it has been achieved only with the more trained prosthesis.

In our participant, the continuative use of multichannel and multi-nerve intraneural stimulation, providing a richer and more pleasant sensory feedback, showed to induce prosthesis embodiment. More importantly, the acute employment of such feedback signals during the test induced an even deeper embodiment compared to non-invasive tactile substitution. However, a comprehensive analysis of both experiments suggests that sensory- and action-oriented embodiment may not always completely match. While the quality of sensory feedback and the degree of human-like appearance of the prosthesis are key factors to attain the former, an operative tool-like embodiment is only achieved through a learning process that leads to proficiency.

## Data Availability Statement

The raw data supporting the conclusions of this article will be made available by the authors, without undue reservation, to any qualified researcher.

## Ethics Statement

The studies involving human participants were reviewed and approved by Campus Bio-Medico Ethics Committee and the Ethics Committee of University of Milano-Bicocca. The patients/participants provided their written informed consent to participate in this study. Written informed consent was obtained from the individual(s) for the publication of any potentially identifiable images or data included in this article.

## Author Contributions

GD, DR, and AMa designed the study, analyzed the data, and wrote the manuscript. CS, AMi, and MD’A performed the study, analyzed the data, and wrote the manuscript. VDe performed the surgery and collaborated during the experiments. VDi designed the study and collaborated during the experiments. RS and LZ collaborated during the experiments. EG supervised the experiments. All authors discussed the results and commented on the manuscript.

## Conflict of Interest

The authors declare that the research was conducted in the absence of any commercial or financial relationships that could be construed as a potential conflict of interest.

## Footnotes

1 https://www.prensilia.com/portfolio/ih2-azzurra/

2 https://www.ossur.com/en-us/prosthetics/arms/i-limb-access
